# A productivist drift of active ageing policies in Italy: regional inequality and economic growth in 2007–2018

**DOI:** 10.3389/fpubh.2026.1940760

**Published:** 2026-10-29

**Authors:** Valerio Intraligi, Francesco Barbabella, Bernard A. Steinman, Mirko Di Rosa, Davide Lucantoni, Giovanni Lamura, Marco Socci, Andrea Principi

**Affiliations:** 1 Centre for Socio-Economic Research on Aging, National Institute of Health and Science on Aging (IRCCS INRCA), Ancona, Italy; 2 Department of Health and Caring Sciences, Linnaeus University, Växjö, Sweden; 3 Family and Consumer Science Department, University of Wyoming, Laramie, WY, United States; 4 Unit of Geriatric Pharmacoepidemiology and Biostatistics, National Institute of Health and Science on Aging (IRCCS INRCA), Ancona, Italy

**Keywords:** active ageing, active ageing index, cumulative disadvantage, policy, positive ageing, productive ageing, public health, regional inequality

## Abstract

**Introduction:**

Active ageing is one of a family of ‘positive ageing’ paradigms—alongside healthy, successful and productive ageing—proposing a multidimensional response to population ageing that ultimately leads to better health, wellbeing and participation in later life. Critical gerontology has long argued, however, that the policy implementation of active ageing drifts towards a narrow productivist focus on later-life employment. Drawing on perspectives from political economy of ageing and place-based cumulative (dis)advantage theory, this study examines whether the multidimensional concept of active ageing is matched by multidimensional implementation across Italian regions, and how regional economic growth is associated with domain-specific trajectories.

**Methods:**

Using an ad-hoc Italian version of the Active Ageing Index (AAI) at the regional (NUTS 2) level over the period 2007–2018, we decompose the contribution of the four AAI domains to overall regional AAI growth, and we estimate associations between changes in real per capita gross domestic product and changes in each domain through cross-sectional and pooled ordinary least squares models, with time-period and region fixed effects as robustness checks, complemented by sensitivity analyses.

**Results:**

The study found that AAI scores rose in all regions, but the employment domain accounted for 75.5% of national growth, confirming a productivist pattern of implementation. Regional economic growth was positively associated with changes in the employment domain and negatively associated with changes in the participation in society domain—a pattern consistent with aggregate-level crowding-out of unpaid participation by paid work rather than with resource expansion. Furthermore, Southern regions combined the lowest AAI growth with the weakest conversion of economic growth into active ageing gains, consistent with place-based cumulative disadvantage.

**Discussion:**

Active ageing development in Italian regions has in practice operated as productive ageing in its narrowest sense. This leads to concerns about whether regional active ageing policies in the 2000s and 2010s substantially contributed to increasing health outcomes, wellbeing and participation of older adults.

## 1 Introduction

### 1.1 Active ageing in Europe

In general, populations around the globe are becoming older on average, and issues related to population ageing are of preeminent concern to policy makers and those who manage and administer programmes that target growing numbers of older people. Globally, Italy is ranked second among countries with more than 1 million people, with the highest estimated share (24.9%) of people aged 65 and over (1). Only Japan has a greater percentage of older adults (30.2%). Longer life expectancies should be considered as a positive outcome, which enable individuals to develop and enjoy relationships with loved ones, and to fulfil personal goals, for more years gained. At the same time, longer life expectancies may also strain societies, including welfare and health care systems, where governing bodies are charged with enacting policy and programmes to promote wellbeing among growing numbers of older adults and a shrinking portion of adult population.

Against this backdrop, the past half-century has seen the emergence of a family of partly overlapping ‘positive ageing’ paradigms that challenge deficit-based accounts of later life: successful ageing (2), productive ageing (3, 4), healthy ageing—now consolidated in the World Health Organization (WHO) Decade of Healthy Ageing 2021–2030 (5)—and active ageing (AA) (6, 7). These frameworks share an emphasis on strengths, participation and wellbeing in later life, yet they differ in scope and in normative orientation, and their boundaries are frequently blurred in research and policy (8, 9). This conceptual instability is not merely terminological: which paradigm implicitly guides policy, determines which later-life activities are promoted, measured and resourced, and for whom.

In recent decades, the European Union (EU) has urged its member states to address population ageing and its consequences through political means. Today, there is broader awareness among policy makers of the practical need for keeping older adults engaged as contributors to their communities, and to the broader society. To facilitate optimal ageing many EU countries have sought to promote AA by focusing greater resources on factors known to support the capacity of older people to remain active and fulfilled contributors to their societies.

Early in its conceptualisation—that is, from the late 1990s until the outset of the 21st century—AA was predominantly oriented towards the unidimensional goal of increasing labour market participation of older adults. Indeed, among the key priorities set out by the EU was the extension of working life by delaying retirement to promote productivity and potentially postpone collection of state-provided pensions (10–13). As a result of these successful efforts, employment rates of older workers (aged 55–64) within the 27 EU countries (EU-27) increased from 43.2% in 2009 to 65.2% in 2024 (14).

The EU has expanded its conceptualisation of AA, in more recent years, to incorporate additional dimensions in the attempt of promoting independence and social engagement among older people across Europe. The multidimensionality of AA has become a distinctive feature (9) of a more holistic conceptualisation that includes areas like quality of life, mental and physical wellbeing, and social participation of older people (15), to be fostered through activities such as lifelong learning, volunteering, and intergenerational practices, among other social domains (16, 17). In 2012, EU-27 policy bodies recognised this multidimensional approach, corresponding with modern conceptualisations of AA, with the establishment of the “European Year for Active Ageing and Solidarity between Generations” (18, 19).

Against this background, the present study asks whether the multidimensional concept of active ageing is matched by multidimensional implementation, and whether regional economic growth promotes active ageing evenly across its domains. We address these questions for Italian regions over the period 2007–2018, using a subnational adaptation of the Active Ageing Index (AAI). Theoretically, we draw on the conceptualisation of AA as defined by the WHO and operationalised by the AAI to investigate possible place-based cumulative (dis)advantage and situate Italian regional inequalities in the opportunity to age actively.

### 1.2 Conceptualising and measuring active ageing

At a conceptual level, there have been two main perspectives of AA. On one hand, the comprehensive version, associated with the WHO (6) framework, encompasses health, participation and security across the life course. The WHO (6) defined it as the process of optimising opportunities for health, participation and security in order to enhance quality of life as people age. On the other hand, the productivist version, dominant in the economic policy agendas of the EU and of the Organisation for Economic Co-operation and Development (OECD), privileges the extension of working lives in response to fiscal pressures on pension and welfare systems (8, 20–22). Under the productivist reading, ‘active’ tends to collapse into ‘economically productive’, and the broader participatory, health-related and environmental dimensions of the concept risk remaining rhetorical.

In any case, the adoption of AA constructs has potential to better inform policies aimed at benefiting both older individuals and society as a whole (23). Comparative evidence regarding AA trends in different countries relies on analytical tools aimed at operationalizing and quantifying the concept. The most used at international level is the AAI (24, 25), which is based on 22 different indicators, across four macro-domains, and ranges between 0 and 100 (the higher the score, the higher the level of AA measured). The aggregation process follows a two-step procedure based on a weighing system (26), according to which domains in *employment* and *participation in society* represent 35% each of the overall score (see Table 1). The remaining 30% of AAI is jointly composed of the *independent, healthy and secure living* domain (10%) and the *capacity and enabling environment for active ageing* domain (20%).

**Table 1:** UNECE Active-ageing index domains and indicators weighting structure.

Domain name	Weight within AAI	Indicator name	Weight within domain
1. Employment	35.0	1.1 Employment rate 55–59	25.0
		1.2 Employment rate 60–64	25.0
		1.3 Employment rate 65–69	25.0
		1.4 Employment rate 70–74	25.0
2. Participation in society	35.0	2.1 Voluntary activities	25.0
		2.2 Care to children, grandchildren	25.0
		2.3 Care to older adults	30.0
		2.4 Political participation	20.0
3. Independent, healthy and secure living	10.0	3.1 Physical exercise	10.0
		3.2 No unmet needs of health and dental care	20.0
		3.3 Independent living arrangements	20.0
		3.4 Relative median income	10.0
		3.5 No poverty risk	10.0
		3.6 No severe material deprivation	10.0
		3.7 Physical safety	10.0
		3.8 Lifelong learning	10.0
4. Capacity and enabling environment for active ageing	20.0	4.1 RLE achievement of 50 years at age 55	33.3
		4.2 Share of healthy life years in the RLE at age 55	23.3
		4.3 Mental well-being	16.7
		4.4 Use of ICT	6.7
		4.5 Social connectedness	13.3
		4.6 Educational attainment	6.7

Source: https://unece.org/population/active-ageing-index. RLE, remaining life expectancy.

However, in some cases measurement of AA through the AAI has been criticised because it does not consider the degree to which older people value or want to be engaged in activities that make up AA constructs (8). Additionally, others have argued that AAI is oriented too much towards a productivist approach (for instance, it does not include any measure of leisure activity) (27). Others pointed to methodological choices conditioned by possible arbitrariness, since domain weights and the selection of indicators were both chosen according to the recommendations of an expert group, rather than based on empirical evidence (27). Despite these criticisms, the AAI has become a widely used operational tool to analyse AA trends within and between countries and take policy decisions (15, 28, 29), leading to concerns on possible biases of such top-down approach that might fail to consider real needs and preferences of older people.

### 1.3 Productivist drift and structural lag

From the perspective of political economy of ageing, the experience of later life is shaped less by individual choices than by the economic and political structures that allocate resources, roles and status across the life course (30). Old-age dependency is not a natural consequence of decline, but is in good part a ‘structured dependency’ created by retirement rules, pension arrangements and labour-market institutions (31). AA policy is therefore not a neutral translation of empirical evidence, but a contested policy discourse whose practical content reflects the priorities of the institutions that promote it (7).

The theory of structural lag offers a complementary mechanism for why policy implementation may diverge from concept. Riley and colleagues (32) argued that social structures and institutions systematically lag behind both demographic change and changing ideas about later life. Opportunity structures for older people’s participation evolve more slowly than the population, and more slowly than the concepts meant to guide policy. Applied to AA, even where the multidimensional concept is formally adopted, the most developed implementation instruments available to governments remain those of labour-market policy, whereas instruments for promoting social participation, lifelong learning or enabling environments are somehow weaker, newer and often devolved to lower levels of government. Measurable change should therefore concentrate where mature instruments exist.

Taken together, the productivist-drift and structural-lag arguments yield a clear empirical expectation: notwithstanding the formal multidimensionality of the AA concept and of the AAI, observed growth in composite measures of active ageing should be driven disproportionately by the employment domain (Hypothesis 1, H1).

### 1.4 Regional inequality and place-based cumulative disadvantage

Concerning inequality in later life, cumulative advantage/disadvantage theory holds that initial advantages and disadvantages compound over the life course, so that heterogeneity and inequality among older people are systematically produced and amplified over time, rather than being randomly distributed (33). Cumulative inequality theory extends this insight by emphasising that such trajectories are generated by social systems—families, markets, institutions—and transmitted across generations (34). While these perspectives were developed primarily at the individual level, research on social exclusion in later life has shown that place is a structuring axis of old-age inequality: the neighbourhood, community and services dimension of exclusion conditions older people’s access to material resources, social relations and civic participation (35).

Extending this reasoning to the regions, they can be conceived as opportunity structures for ageing actively: their labour markets, service infrastructures, transport, civic traditions and associational density translate macro-level processes into unequal local chances of remaining employed, volunteering, caring or participating in community life. A territorial reading of cumulative (dis)advantage then follows: regions that accumulate enabling conditions—wealth, services, social capital—progressively widen their residents’ opportunities to age actively, while lagging regions accumulate barriers, in a self-reinforcing dynamic consistent with the unevenness predicted by structural lag.

Such territorial stratification is particularly salient in welfare systems of the Southern European, familialist type, in which formal services are comparatively weak, the family absorbs a large share of care responsibilities, and social policy competencies are strongly regionalised (36). In Italy, where health and most social participation-related policies are designed and managed by regional governments (37), the region is precisely the scale at which enabling environments for AA are produced—or fail to be produced.

Effectively monitoring and evaluating AA outcomes and determinants requires acknowledgement of geographical contexts that are often shaped by socioeconomic gaps and/or inequalities in resource endowments across territories. Cultural and traditional aspects of regional diversity may also play a major role in shaping preferences of individuals over whether to age actively or not (38), and in which way (e.g., by being involved in the labour market, volunteering, grand parenting, and so on).

It is currently unclear whether in wealthier Italian regions (i.e., those with more resources) AA rates are higher than in the other regions, and whether regional differences in economic resources reflect regional differences across various AA domains. Under a resource-expansion (complementarity) account, economic growth increases the resources available to older people and to their territories—e.g., income, health and social services, civic infrastructure—and should therefore be associated with gains across all AA domains. This is consistent with evidence that employment and volunteering can complement each other (39, 40). Under a substitution (crowding-out) account, by contrast, time is the binding constraint: because paid work absorbs time and energy that would otherwise flow to unpaid activities (41–43), growth episodes that draw older adults into—or retain them in—employment should be accompanied by contractions in volunteering, informal care and other forms of social participation at the aggregate level.

The employment domain is indeed expected to co-move positively with regional growth under both accounts. Instead, the relation between economic growth and participation in society domain leads to two competing hypotheses: a positive association with growth supports resource expansion (Hypothesis 2a, H2a), whereas a negative association supports aggregate crowding-out (Hypothesis 2b, H2b).

The independent, healthy and secure living domain and the capacity and enabling environment domain largely comprise slow-moving structural components—e.g., life expectancy, health status, educational attainment, poverty risk—which are expected to respond to long-term trends and policy rather than to short-run regional growth, consistently with the structural-lag argument set out above. We accordingly expect weak associations with regional growth over the periods observed here, and read such weakness as an indication that adjustment in these two domains operates on a longer temporal scale, rather than as an indication that economic resources are irrelevant to them (Hypothesis 3, H3).

### 1.5 The Italian case and aim of the study

In the European context, Italy represents an interesting case of regional diversity. Since the foundation of the Italian state in 1861, its territorial divide is among the most prominent barriers to national development (44, 45). According to Principi and colleagues (15), the socio-economic gap between Northern and Southern Italian regions is appreciable in several indicators related to population, wealth and quality of life. Figure 1 provides graphical insight into the economic gap, plotting differences in the value of per capita gross domestic product (GDP) in 2007 (inset a.) and the growth observed by each region in GDP between 2007 and 2018 (inset b).

**Figure 1:**
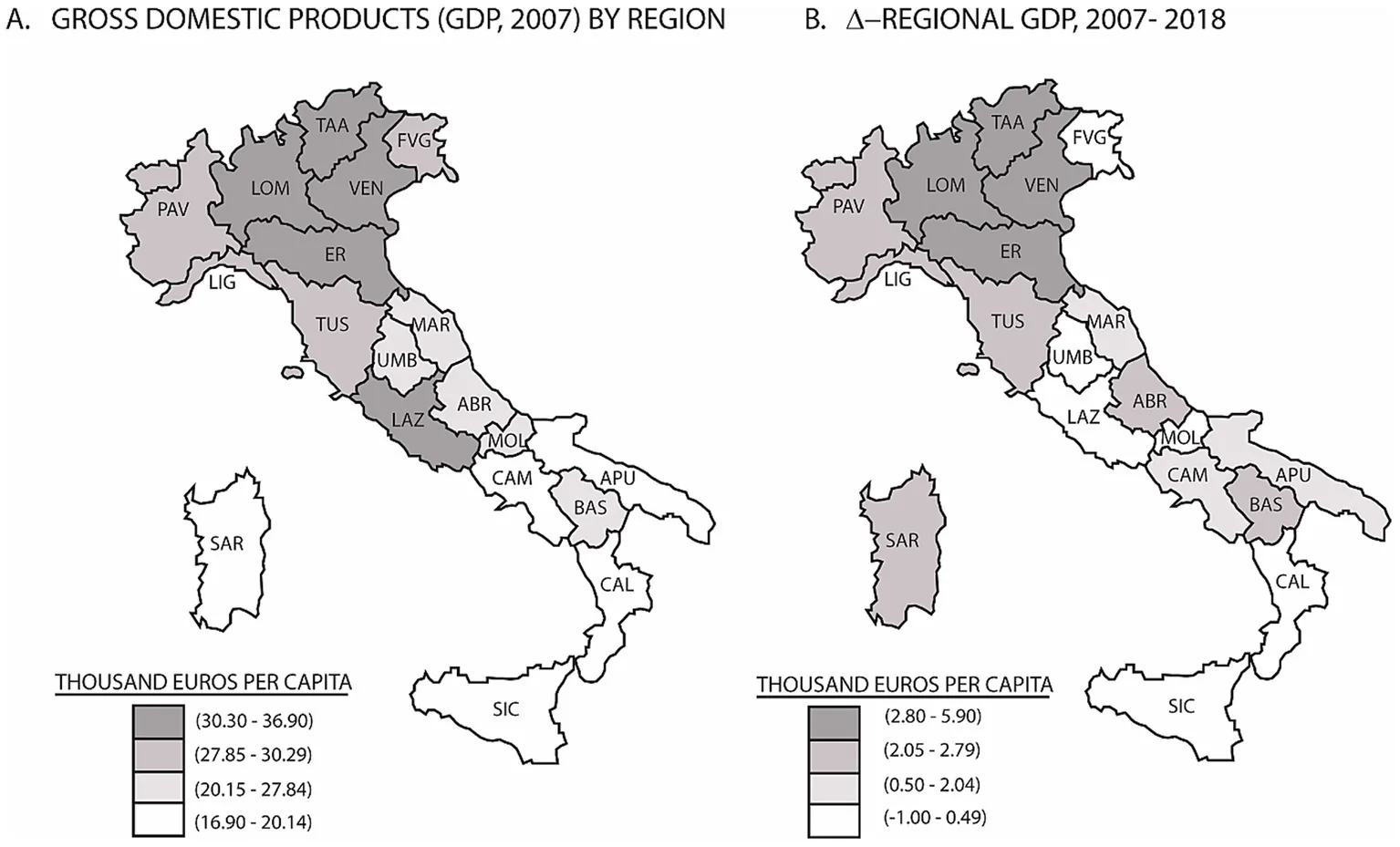
**(A)** GDP *Per Capita* by Italian Region (2007); and **(B)** Change in Regional GDP Over Time (2007–2018). Source: Authors’ calculations on Eurostat regional database data. https://ec.europa.eu/eurostat/web/regions/data/database. For data availability reasons, Piedmont and Aosta Valley regions are grouped in a single region. PAV, Piedmont & Aosta Valley; LOM, Lombardy; TAA, Trentino Alto Adige; VEN, Veneto; FVG, Friuli Venezia Giulia; LIG, Liguria; ER, Emilia Romagna; TUS, Tuscany; UMB, Umbria; MAR, Marche; LAZ, Latium; ABR, Abruzzo; MOL, Molise; CAM, Campania; APU, Apulia; BAS, Basilicata; CAL, Calabria; SIC=Sicily; SAR, Sardinia.

As depicted in Figure 1, the relative economic disparities between Italian regions shifted minimally during the period analysed in this study (2007–2018). Whereas four Northern regions (i.e., Trentino-Alto Adige, Lombardy, Veneto and Emilia-Romagna) continuously exhibited the highest values in GDP per capita, compared to Southern and Central Italy (i.e., Umbria, Latium, Molise, Calabria and Sicily), which experienced limited increases (or even a decreases) in regional GDP per capita during the study timeframe.

The general aim of this study is to understand whether and how multidimensionality of AA is substantially addressed by policy makers throughout Italian regions, and whether and how regional diversity in economic growth may affect participation in various AA domains. Previous literature on AA has rarely focused directly on assessing and comparing trends of AA multidimensionality, at least from a dynamic perspective (15, 46). Additionally, there is the need to empirically demonstrate whether the multidimensional conceptualisation of AA has produced relevant outcomes, or whether the policy impact still concerns one or more specific AA domains.

Thus, the first objective of this study is to investigate, from a regional perspective, the extent to which four different AA domains (from the AAI)—i.e., (1) *employment*, (2) *participation in society*, (3) *independent, healthy and secure living*, and (4) *capacity and enabling environment for active ageing—*impact overall changes in AA over time (2007–2018). In line with H1, we expect changes in the employment domain to dominate overall AAI growth.

The second objective of the study is to investigate whether regional economic growth plays a role in explaining regional trends in different AA dimensions. Indeed, while associations between economic growth and participation of older workers in the labour market have been noted (47), the literature on AA provides only limited evidence about the relationship between income-growth and older adults’ participation in other aspects of society (e.g., through forms of social participation, caregiving, grandparenting, volunteering). Here, the competing hypotheses H2a (resource expansion) and H2b (aggregate crowding-out), as well as the hypothesis H3 (no effect on other AA dimensions) are put to the test.

## 2 Materials and methods

### 2.1 Data

This study employed an ad-hoc version of the AAI, constructed using Italian national data sources from the Italian National Institute of Statistics (ISTAT). Indeed, the original AAI has been calculated for EU countries [e.g., (48, 49)] by using European data sources. In some cases, the index was further developed with national sources (49), and applied at the subnational level [e.g., (15, 29, 46, 50–52)]. The importance of investigating AA at the subnational level has been emphasised, due in part to existing within-country diversity and differences in cultural, social and structural contexts (9, 38, 53).

In particular, the adapted Italian AAI used in this study aligns with definitions and concepts of the original AAI (AAI-EU). Of the total 22 indicators, 13, sourced from the Statistic on Income and Living Conditions (SILC), Italian Labour Force Survey (IT-LFS) and mortality tables, were identical to standard AAI-EU items. To obtain the remaining nine indicators at the regional level (which was not possible by using the same sources of the standard AAI-EU), data were derived following guidelines directing the calculation of the AAI on a subnational level (49) from Italian ISTAT surveys on Aspects of Daily Life (AVQ), Family and Social Subjects (FSS), and the European Health Interview Survey (EHIS). As a result, some discrepancies from the original AAI-EU were observed in indicators 2.2 and 2.3 (see Supplementary Table A2), and particularly when specific questions were not released by the ISTAT (this affected indicators 3.7 and 4.4). Nevertheless, all 22 indicators were available at the regional level. The Italian AAI, employed in this study, maintains the same weights as the original AAI. To allow for the analysis of trends in the Italian AAI, we used data for four different time-points (i.e., 2007, 2009, 2012 and 2018). Indicators for some years were imputed using data from immediately previous or subsequent years, according to the availability of each data source [see (54)]. The 2007–2018 window thus provides a consistent pre-pandemic baseline of active ageing dynamics across Italian regions: pandemic-period disruptions affected several AAI indicators in ways that are still being assessed (55), and this baseline constitutes the benchmark against which the implementation of Italy’s forthcoming National Plan for Active Ageing can be evaluated.

### 2.2 Key independent variable

We used regional economic diversity as key independent variable. We operationalised regional economic diversity by using a measure of economic development, i.e., the observed change in regional gross domestic product (GDP) per capita by Nomenclature of Territorial Units for Statistics (NUTS) 2 regions at inflation-adjusted amounts. We relied on publicly available data from the Eurostat regions database (see https://ec.europa.eu/eurostat/web/regions/data/database).

Although Italy is composed of 20 distinct regions, several ISTAT regional databases used in this study combine Italy’s smallest region Aosta Valley (ca. 126,000 inhabitants) with Piedmont (a larger bordering region) for statistical needs. We therefore use the same aggregation, totalling 19 regional units. Because the two units differ markedly in demographic size, the values of this aggregated unit reflect Piedmont to a much greater extent than Aosta Valley; the same aggregation applies to all the analyses reported below.

### 2.3 Dependent variables

The AAI is composed of four different domains aggregated by means of a weighting system (see Table 1). In this study, we assessed regional trends in AA multidimensionality. Hence, as a response variable, we use unweighted changes in time in each of the four different AAI domains, separately considered.

### 2.4 Analyses

To assess the effects of different AAI domains in explaining overall AAI trends in Italian regions, we performed a decomposition of growth in each region’s AAI by single domains over the period 2007–2018. In particular, we calculated the percentage contribution of changes in each domain score to the overall AAI change for each region.

After describing the contributions of each domain in relation to the overall AAI change during the study period (2007 to 2018), we explored possible associations between change in regional GDP per capita (as a proxy for differences in regional development) and changes in four different AAI domains within the same period. We applied standard ordinary least squares (OLS) regression, according to the following econometric specification:

$$\Delta D_{r,2007-2018}=\alpha +\beta_{1}\text{ΔPCGD}P_{r,2007-2018}+\varepsilon$$ (1)

Where $\Delta D_{r,2007-2018}$ is the growth in the reference domain in region *r* over the period *2007* to *2018* and $\text{ΔPCGD}P_{r,2007-2018}$ is the *2007* to *2018* growth in per capita GDP in region *r*.

Then, in order to maximise information, we exploited the dynamic structure of the data and estimated a pooled OLS regression model. In particular, we pooled variations over three different time-periods (2007–2009, 2009–2012, and 2012–2018), according to the following equation:

$$\Delta D_{r,t_{0}-t_{1}}=\alpha +\beta_{1}\text{ΔPCGD}P_{r,t_{0}-t_{1}}+\varepsilon$$ (2)

Where $\Delta D_{r}$ is the growth in the reference domain in region *r* over the period *t0-t1* and $\text{ΔPCGD}P_{r,t_{0}-t_{1}}$ is the *t0-t1* growth in per capita GDP in region *r*.

Finally, we carried out a check to test the robustness of results from the previous univariate analyses. Specifically, we estimated an augmented version of Equation 2 considering time period-effects that capture differences between periods that are common to all regions, as follows:

$$\Delta D_{r,t_{0}-t_{1}}=\alpha +\sigma_{t}+\beta_{1}\text{ΔPCGD}P_{r,t_{0}-t_{1}}+\varepsilon$$ (3)

Where the term $\sigma_{t}$ represents time-period effects.

To attenuate possible omitted-variable bias, we augmented Equation 3 with region fixed effects, so that the coefficient of interest is identified only by within-region variation across the three time-periods, as follows:

$$\Delta D_{r,t_{0}-t_{1}}=\alpha +\sigma_{t}+\gamma_{r}+\beta_{1}\text{ΔPCGD}P_{r,t_{0}-t_{1}}+\varepsilon$$ (4)

Where the term $\gamma_{r}$ represents region effects, which absorb time-invariant regional characteristics potentially correlated with both domain changes and per capita GDP growth. Since the sample covers 19 regions observed over three time-periods, this last specification is demanding in terms of degrees of freedom. In all models reported in Table 2 and Supplementary Table A3, observations are weighted by each region’s share of the national population in 2007, and standard errors are robust and clustered at the regional level (19 clusters), so as to account for the non-independence of the repeated observations available for each region. Given the aggregate, observational design of the study, all estimates are interpreted as regional-level associations rather than causal effects, and no inference is drawn about individual-level behaviour (56).

**Table 2:** AAI domains change and regional economic growth by NUTS 2 regions (Pooled OLS: 2007–2009/2009–2012/2012–2018).

	(1)	(2)	(3)	(4)
	Δ Employment domain	Δ Participation domain	Δ Indep. living domain	Δ Capacity domain
Panel A				
$\text{ΔPCGD}P_{r,t_{0}-t_{1}}$	1.485***	−0.200**	−0.114	0.578***
	(0.107)	(0.069)	(0.072)	(0.120)
*Period FE*	no	no	no	no
*Region FE*	no	no	no	no
*R^2^*	0.738	0.132	0.016	0.329
Panel B				
$\text{ΔPCGD}P_{r,t_{0}-t_{1}}$	0.694**	−0.348**	−0.242	0.115
	(0.205)	(0.108)	(0.172)	(0.200)
*Period FE*	yes	yes	yes	yes
*Region FE*	no	no	no	no
*R^2^*	0.857	0.471	0.055	0.525
Panel C				
$\text{ΔPCGD}P_{r,t_{0}-t_{1}}$	0.484*	−0.473*	−0.428	0.226
	(0.219)	(0.187)	(0.320)	(0.339)
*Period FE*	yes	yes	yes	yes
*Region FE*	yes	yes	yes	yes
*R^2^*	0.938	0.592	0.124	0.589

Robustness checks. Pooled OLS regressions. *N* = 57 (19 regions × 3 time-periods). Robust standard errors clustered by region. Models weighted by regions’ share of national population in 2007. For data availability reasons, Piedmont and Aosta Valley regions are grouped in a single region. * *p* < 0.05, ** *p* < 0.01, *** *p* < 0.001. In Panel C, wild cluster bootstrap-t p-values for the coefficient on Δ GDP per capita, computed with the null imposed and Webb weights (9,999 replications), are 0.020, 0.030, 0.195 and 0.560 in columns 1 to 4, and the corresponding 95% confidence sets are [0.066; 0.898], [−0.771; −0.067], [−1.019; 0.222] and [−0.370; 0.945].

## 3 Results

We based decomposition of the relative contribution of single domains to the overall regional AAI change in each Italian region on AAI breakdowns by domain scores in 2007 and 2018. Accordingly, Table 3 shows rankings of overall change in AAI, and variation in AAI between 2007 and 2018 following the decomposition of AAI into its four domain components, along with the relative percent contribution of each domain to total AAI change. Thus, for each row in Table 3, the sum of values reported in the last four columns are equal to 1, making it possible to identify domains that contribute more or less to overall regional AAI change.

**Table 3:** Percentage contribution of AAI domains to AAI change by NUTS 2 regions (2007–2018).

AAI		Overall score		Percentage contribution of domains on AAI change			
Region	Change	2007	2018	Employment	Participation	Independent Living	Capacity
Friuli-Venezia Giulia	6.99	27.36	34.35	0.748	0.087	0.034	0.131
Latium	6.43	27.10	33.53	0.662	0.127	0.051	0.160
Tuscany	6.25	28.41	34.66	0.803	0.049	0.046	0.102
Trentino-Alto Adige	6.24	30.95	37.19	0.748	0.08	−0.005	0.177
Lombardy	6.16	27.94	34.10	0.769	0.085	0.049	0.096
Molise	6.16	25.82	31.98	0.725	0.037	0.034	0.204
Marche	6.02	27.32	33.35	0.732	0.16	0.041	0.067
Veneto	6.00	27.75	33.75	0.801	0.007	0.049	0.143
Abruzzo	5.82	26.42	32.24	0.730	0.103	0.062	0.105
Emilia-Romagna	5.79	29.12	34.91	0.771	0.078	0.047	0.104
Piedmont and Aosta Valley	5.76	27.07	32.83	0.872	0.062	0.051	0.015
Basilicata	5.52	26.39	31.91	0.626	0.159	0.050	0.166
Liguria	5.37	27.76	33.13	0.851	0.063	0.037	0.048
Umbria	5.36	27.39	32.75	0.802	0.151	−0.03	0.076
Italy	5.36	26.98	32.33	0.755	0.088	0.043	0.114
Sardinia	5.26	26.25	31.52	0.786	0.006	0.054	0.154
Apulia	4.48	24.60	29.08	0.598	0.178	0.064	0.160
Calabria	3.57	25.28	28.85	0.594	0.251	0.004	0.152
Sicily	3.57	24.29	27.86	0.622	0.132	0.026	0.210
Campania	3.21	25.09	28.30	0.810	−0.036	0.081	0.145
Weighted mean	5.35	26.90	32.33	0.746	0.083	0.047	0.124

Regions ranked by AAI change score over 2007–2018. Mean values exclude aggregate data for Italy and are weighted by regions’ share of national population in 2007. For data availability reasons, Piedmont and Aosta Valley regions are grouped in a single region.

According to Table 3, the observed changes in overall AAI from 2007 to 2018 were positive in each Italian region, indicating that AAI scores increased throughout the country during that time. At the national level, the mean change in AAI score was Δ5.36—ranging among regions from a low of Δ3.21 (Campania) to Δ6.99 (Friuli-Venezia Giulia). In all regions, the *employment* domain contributed most to overall AAI change, nationally accounting for 75.5% of the total AAI increase—ranging from 59.4% (Calabria) to 87.2% (Piedmont and Aosta Valley). On average, the *capacity and enabling environment for active ageing* domain contributed the next greatest amount to AAI change (national value, 11.4%)—ranging from 1.5% (Piedmont and Aosta Valley) to 20.4% (Molise). Also, 8.8% of AAI growth nationally was attributed to the *participation in society* domain, ranging from −3.6% (Campania) to 25.1% (Calabria). As for the *independent, healthy and secure living* domain, it explained only 4.3% of AAI growth nationally, ranging from −3% (Umbria) to 8.1% (Campania). These results support H1: notwithstanding the multidimensional architecture of the index, realised growth in active ageing across Italian regions was overwhelmingly employment-led.

To investigate the extent to which single AAI domains reflect differences in regional economic development, we assessed changes in each region’s GDP per capita income using four distinct univariate OLS regression models, following Equation 1. Scatterplots and OLS regression lines of the four different univariate relationships—i.e. between the change in each of the four AAI domains by region (Y axis) and the change in each region’s GDP per capita (X-axis) between 2007 and 2018—are shown in Figure 2.

**Figure 2:**
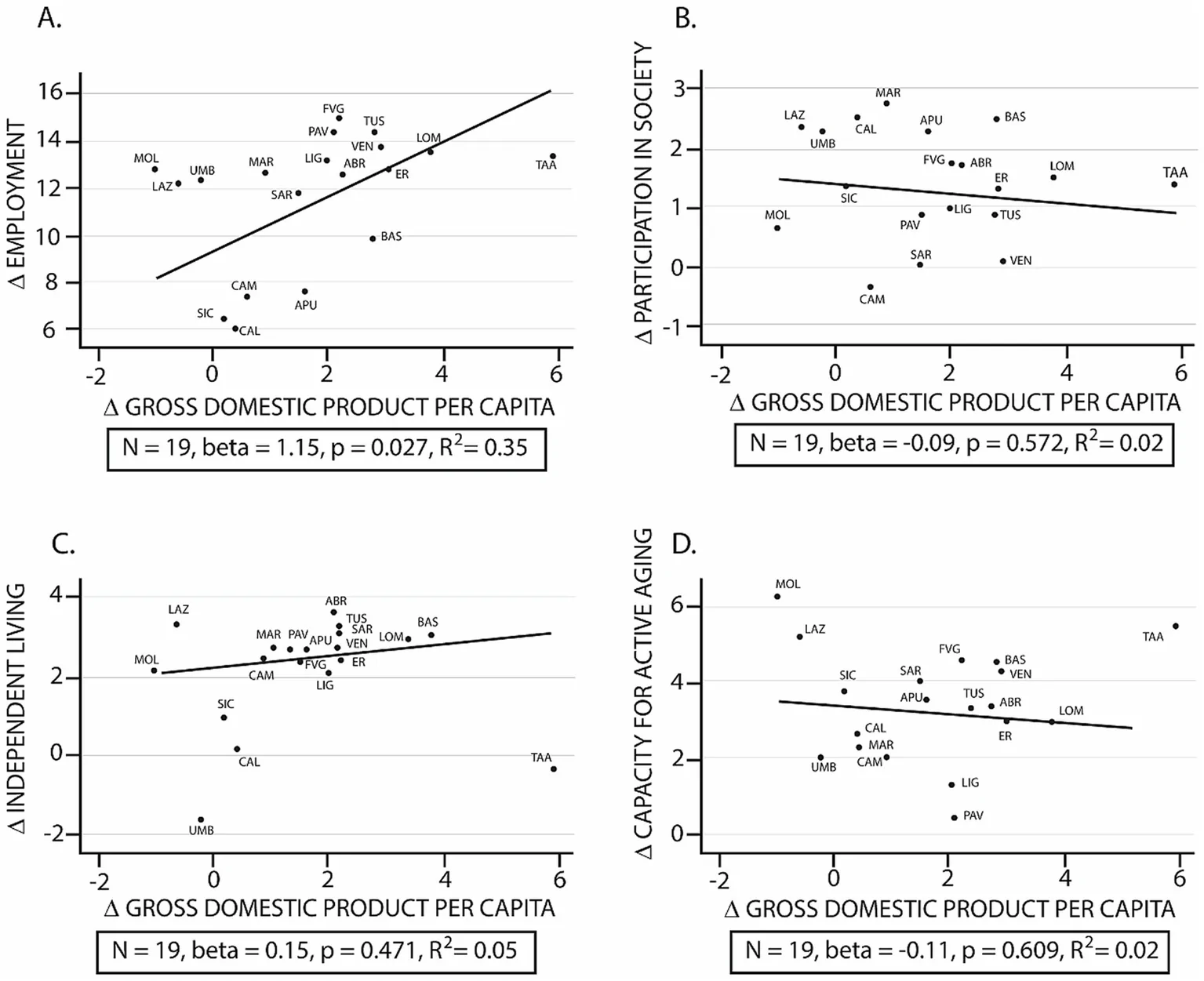
AAI Domains Change and Regional Economic Growth, by NUTS 2 Regions (OLS: 2007–2018). Univariate Scatterplots and Regression lines (four panels: **A** - Employment; **B** - Participation in society; **C** - Independent living; **D** - Capacity for active ageing). OLS univariate regressions. *N* = 19. Robust standard errors. Models weighted by regions’ share of national population in 2007. For data availability reasons, Piedmont and Aosta Valley regions are grouped in a single region. PAV, Piedmont & Aosta Valley; LOM, Lombardy; TAA, Trentino Alto Adige; VEN, Veneto; FVG, Friuli Venezia Giulia; LIG, Liguria; ER, Emilia Romagna; TUS, Tuscany; UMB, Umbria; MAR, Marche; LAZ, Latium; ABR, Abruzzo; MOL, Molise; CAM, Campania; APU, Apulia; BAS, Basilicata; CAL, Calabria; SIC, Sicily; SAR, Sardinia.

As depicted in Figure 2A, and as expected, the measure of regional development (i.e., variations in GDP per capita) was positively and with statistical significance associated with changes in the *employment* domain (*p* = 0.027), with the model explaining more than one-third of the total variation in the change in this domain (*R*^2^ = 0.35). In general, higher regional economic growth reflects higher regional employment rates. Nevertheless, Figure 2A also highlights interesting elements of regional diversity. For instance, on the left side of the figure (above the regression line) we mostly display regions in Central Italy, such as Latium, Umbria and Marche. Thus, these regions experienced appreciable increases in the *employment* domain even in the presence of smaller increases (or even decreases) in GDP per capita. By contrast, Southern regions lying far below the regression line (including Sicily, Apulia, Campania and Calabria) though experiencing changes in GDP per capita analogous to those observed for central regions, experienced considerably lower increases in the *employment* domain. Figures 2B–D reveal no significant relationship between the remaining three AAI domains (i.e., *participation in society*, *independent, healthy and secure living,* and *capacity and enabling environment for active ageing*), and change in GDP.

To provide richer evidence about the associations under investigation, Figure 3 depicts similar analyses over three different sub-periods available in our data (i.e., 2007–2009, 2009–2012, and 2012–2018; see Equation 2) to analyse trends in changes in GDP per capita and their associations with AAI domain scores, over time. This approach allowed observing short-run variations, in addition to single variation over time, thus increasing data resolution. Since the variables are measured over three different periods, marker labels in Figure 3 indicate the period, in chronological order (i.e., periods 1, 2 and 3) next to each Region.

**Figure 3:**
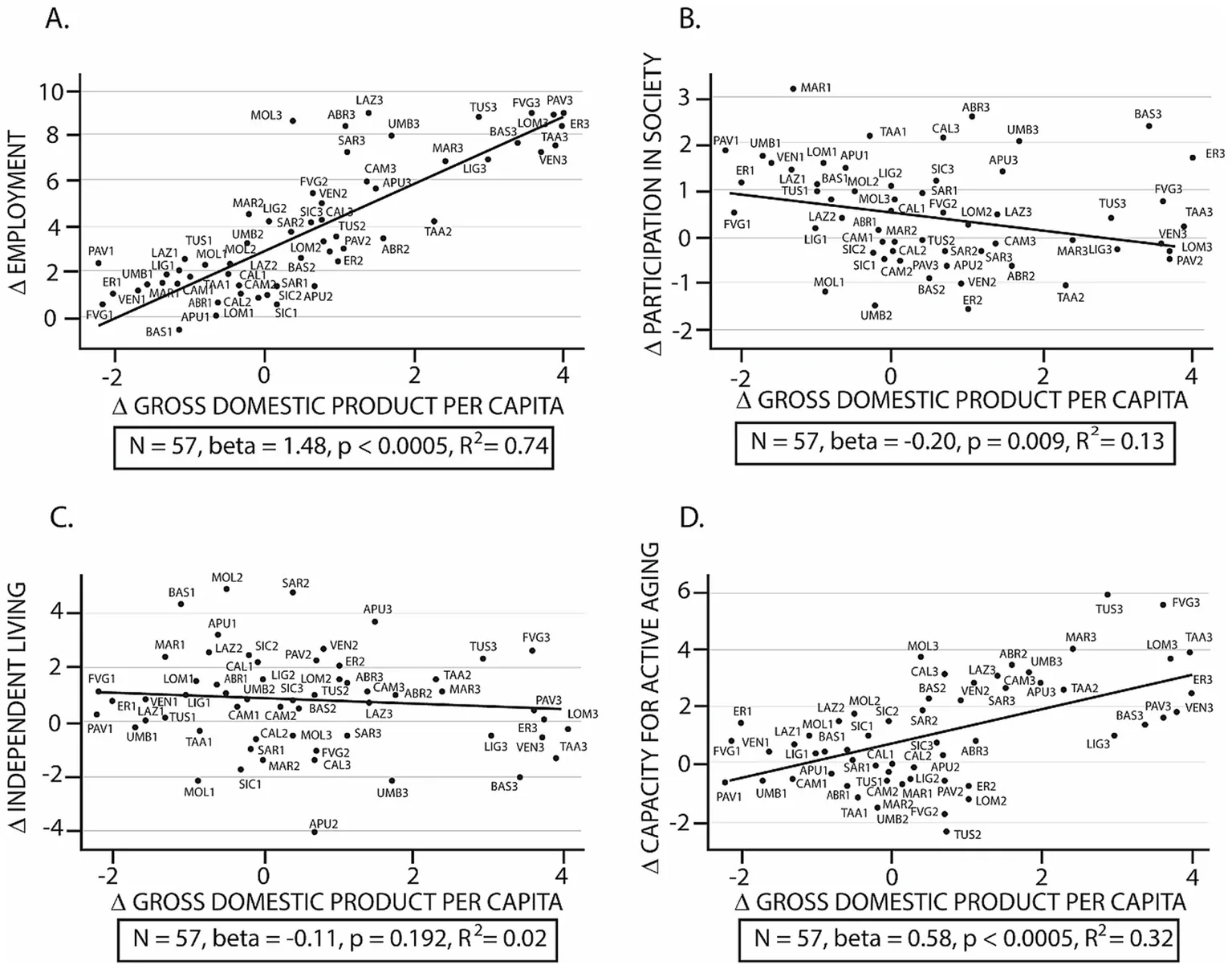
AAI Domains Change and Regional Economic Growth, by NUTS 2 Regions (Pooled OLS: 2007–2009/2009–2012/2012–2018). Univariate scatterplots and regression lines (four panels: **A** - Employment; **B** - Participation in society; **C** - Independent living; **D** - Capacity for active ageing). Pooled OLS univariate regressions. *N* = 57 (19 regions × 3 time-periods). Robust standard errors. Models weighted by regions’ share of national population in 2007. For data availability reasons, Piedmont and Aosta Valley regions are grouped in a single region. PAV, Piedmont & Aosta Valley; LOM, Lombardy; TAA, Trentino Alto Adige; VEN, Veneto; FVG, Friuli Venezia Giulia; LIG, Liguria; ER, Emilia Romagna; TUS, Tuscany; UMB, Umbria; MAR, Marche; LAZ, Latium; ABR, Abruzzo; MOL, Molise; CAM, Campania; APU, Apulia; BAS, Basilicata; CAL, Calabria; SIC, Sicily; SAR, Sardinia. Standard errors in the figure are heteroskedasticity-robust. Estimates of the same models with standard errors clustered by region, on which inference in the paper is based, are reported in Table 2, Panel A.

As shown in Figure 3A, we still noted a positive association between GDP per capita growth and changes in the *employment* domain (*p* < 0.0005). Moreover, the model explained nearly three quarters of the total variation in the change in the *employment* domain (*R^2^* = 0.74). This plausibly depends on the fact that both variables are lower in the first period and higher in the third period due in part to the Great Recession that affected Italy during the period of 2007 to 2011—while real GDP started to increase again during the period of 2012 to 2018, particularly after 2015. The left-hand side margin of each graph displayed in Figure 3 also highlights this feature, in that we mainly see observations of the first period, while on the right-hand side, we mostly see observations of the third period.

According to Figure 3B, showing results for the *participation in society* domain, changes in that domain correlate negatively with economic growth. In particular, the model explains about 13% of the total variation in changes in the *participation in society* domain (*R^2^* = 0.13), while the negative coefficient on GDP per capita growth in this setting is statistically significant at the 1% level (*p* = 0.009). This pattern is consistent with the aggregate crowding-out hypothesis (H2b) rather than with the resource-expansion hypothesis (H2a).

As depicted in Figure 3C, showing results for the *independent and secure living* domain, we detected no significant results. By contrast, Figure 3D, showing results for the *capacity for active ageing* domain, indicates a strong relationship between the explanatory variable and changes in this domain—explaining up to almost one-third of the total variation (R^2^ = 0.32), with a highly significant positive coefficient (*p* < 0.0005). However, caution is needed in interpreting these results, as time-period effects, once included in the model, may capture a substantial part of the total variation in the response variables (therefore, possibly influencing the estimates obtained here). Thus, in the final step of the analysis, we include time-period dummies as additional explanatory variables to capture changes that—between different time—are common to all regions, removing them from the estimated relationship. In this case, in period of recession/expansion they capture the decrease/increase in GDP per capita growth that is common to all regions.

We report in Table 2 both coefficients from univariate models and the coefficients estimated when including time-period dummy variables (Table 2, Panel B) according to the specification in Equation 3. In addition, in Panel C we account for time-invariant unobserved regional characteristics by also entering region fixed effects in the specification, as in Equation 4. The pooled univariate estimates displayed in Figure 3 correspond to Panel A of Table 2, where they are reported with cluster-robust standard errors. Because the number of clusters is small, inference on the coefficient of interest in Equation 4 was additionally checked by means of the wild cluster bootstrap-t with the null imposed and Webb weights (9,999 replications), as recommended for settings with few clusters; the resulting *p*-values and confidence sets are reported in the notes to Table 2. We conducted these analyses to facilitate comparison between estimates obtained when time-period and region effects were included and not included.

When time-period dummy variables are included, variation explained by each model increases substantially, as demonstrated by comparing corresponding R^2^ measures between Panel A and Panel B of Table 2, while a further increase follows the inclusion of region fixed effects in Panel C. This increase concerns the share of variance absorbed by the time-period and region effects, and is not informative about the precision of the coefficient of interest, which decreases across the three panels. Additionally, Panel B indicates that both the employment and participation in society domains each robustly reflected effects previously detected by univariate models, when time-period dummies were included. With reference to the employment domain (column 1)—though decreasing by more than half (1.48 vs. 0.69)—the positive coefficient is still significant at the 1 per cent level, analogously to the negative estimate on the participation in society domain (column 2). Conversely, in the capacity and enabling environment for active ageing domain (column 4), the previously significant effect is no longer present, decreasing substantially from 0.58 to 0.11. This result suggests that the passage of time primarily drives observed changes in this domain, as indicated by the positive and highly significant coefficient on the third-period dummy (not shown in Table 2 due to space constraints). Lastly, still, in the independent, healthy and secure living domain (column 3), no significant correlation was observed.

The estimates in Panel C suggest that these patterns are not driven by time-invariant regional characteristics. In the employment domain (column 1) the coefficient declines further (0.69 vs. 0.48) but remains significant at the 5 per cent level, while in the participation in society domain (column 2) the negative estimate increases in absolute size (−0.35 vs. −0.47) and is also significant at the 5 per cent level. The coefficients on the independent, healthy and secure living domain and on the capacity and enabling environment domain (columns 3 and 4) remain not significant. Overall, the employment and participation in society domains retain a significant association with regional economic growth once both time-period and region fixed effects are included, while the association detected by the univariate model in the capacity and enabling environment domain does not hold. The restriction of the estimates to within-region variation, however, reduces estimates precision, as indicated by the standard errors, which are larger in Panel C than in Panel B in all four domains. The wild cluster bootstrap-t *p*-values reported in the notes to Table 2 confirm the pattern: 0.020 for the employment domain and 0.030 for the participation in society domain, against 0.195 and 0.560 for the two remaining domains. The bootstrap confidence sets for the two former domains, [0.066; 0.898] and [−0.771; −0.067], exclude zero, while those for the independent, healthy and secure living domain and for the capacity and enabling environment domain, [−1.019; 0.222] and [−0.370; 0.945], are wide and include it, which indicates that the absence of a detectable association in these two domains reflects the imprecision of the estimates rather than a coefficient close to zero.

Finally, in Supplementary Table A3 we report two different sensitivity analysis checks for results displayed in Panel C. First, we run two regressions for the Participation domain by progressively leaving out the Calabria and Molise regions, which show extreme domain contributions in Table 2. Second, we run two regressions for the Independent Living and the Capacity domains by leaving out indicators 3.7 and 4.4, respectively, for the reasons already outlined in Section 2.1. Results obtained do not substantially differ from those presented here.

## 4 Discussion

The first objective of this study was to assess the extent to which older adults’ participation in every AA domain has contributed to changes over time in AAI scores of Italian regions. Our results suggest that policy efforts to promote AA in Italy in 2007–2018 had mostly focused on improving participation in the labour market of older people, confirming H1. Orientation towards the *employment* domain is highly influenced by a number of national pension system reforms and interventions introduced in recent decades. Indeed, reforms have been developed and implemented to encourage workers to remain in the labour market up to or beyond the current pension age in Italy, which is 67 years for both men and women, while other policies aimed to tighten up requirements needed to access early-retirement (57, 58). Nevertheless, with respect to the *employment* domain, policy makers at the regional level have relatively limited regulatory power (since these aspects are regulated at the central governmental levels). This is precisely the pattern anticipated by H1. The multidimensional rhetoric of AA coexists with an implementation infrastructure—pension and labour-market levers—whose mature instruments remain overwhelmingly productivist, so that measurable gains concentrate where instruments exist (20, 22, 32). In other terms AA as implemented in Italy has in practice approximated active ageing in its narrowest, employment-centred sense.

In relation to the relevance of regional diversity (46, 59), a second objective of this study was to investigate the extent to which differences in regional trends of AAI domains relate by controlling for regional economic development. We operationalised this concept by using regional per capita GDP growth, with the understanding that regions largely depend on their capacity to exploit and mobilise their own assets to contribute to national performance indicators (60, 61). The main goal was to provide evidence regarding links between regional economic growth and trends in different AA domains, particularly concerning the *participation in society* domain (which policymakers regulate, primarily, at the regional level).

In the case of the *employment* domain, our results document—perhaps not surprisingly—a strong positive link between per capita GDP growth and labour market participation among older adults (and in general, the association is stronger moving from Southern to Northern regions of Italy).

As for the *participation in society* domain, regions experiencing higher economic growth experienced on average lower increases in this domain, confirming H2b. The literature is relatively scarce in describing the effects of income-growth on volunteering and/or other unpaid activities. Veal and Nichols (62) addressed the relationship between inequality and volunteering from a cross-national perspective. Despite documenting greater levels of volunteering in high-income countries, these authors provided little evidence concerning the effects of income growth on volunteering. The results of the present study seem to indicate that the relationship between income growth and social engagement needs further exploration.

On the other hand, our results suggest the possibility that *participation in society* relates to higher levels of social need in more disadvantaged regions—as suggested by lower economic performance in regions with higher growth in the *participation in society* domain (particularly in Southern regions). Indeed, relatively disadvantaged economic conditions can translate not only into reduced wellbeing, but also into weaker development of welfare measures and, therefore, a decreased ability to deal with emerging needs. Encouraging participation of older people in activities such as volunteering and informal caregiving may be a mechanism for policy makers to address these disparities (3, 4). This pattern can also be read from a perspective of cumulative disadvantage and old-age exclusion as informal participation substituting for weak formal provision: in regions where familialist welfare arrangements and thinner service infrastructures prevail (36), unpaid activities such as informal care may expand to meet needs that better-resourced regions absorb through formal services (35).

As for the combined evidence of a positive association between economic growth and the *employment* domain and of a negative relationship with the *participation in society* domain, our results may suggest the existence of a crowding-out effect. Indeed, increases in the *employment* domain may arguably suggest greater time allocated by older adults to paid activities at the expense of unpaid endeavours (41–43, 63). The concept that increased paid employment “crowds out” opportunities to participate in other AA domains is also consistent with results obtained in studies focusing on work restrictions faced by informal family caregivers in Italy (64). Previous research does not reach full consensus on this issue. Indeed, while some studies show that participation in the labour market and engagement in volunteering activities constitute substitutes (65, 66), other analyses provide evidence that participation in such activities complement each other (39, 40). Obviously, ours is an aggregate-level association: it indicates that regional growth episodes are accompanied by opposite movements in paid and unpaid participation, but it cannot establish that the same individuals substitute one activity for the other (56). The crowding-out reading and the compensatory-participation reading discussed above are both consistent with the aggregate evidence, which does not separate them; we therefore report the former as one plausible mechanism rather than as the interpretation favoured by the data.

No significant associations were found between the economic growth and, respectively, the *independent and secure living* domain and the *capacity for active ageing* domain, consistently with H3. Following the reading proposed above, this is not evidence that these two domains are unrelated to economic resources, but that their adjustment operates over a horizon longer than the one spanned by the periods observed here.

Many previous studies have demonstrated the advantages of AA behaviours and opportunities to ageing individuals, in relation to their improved physical and mental health, social engagement, and wellbeing (67). Thus, the promotion of a AA is an important policy aim for ameliorating social and economic challenges linked to population ageing. A central feature of AA is its multidimensionality. In early phases of policy implementation of AA strategies in Europe, assessments have largely neglected its multidimensional facets, and policies have focused almost exclusively on participation of older adults in the labour market (10, 13). More recently, interest by policy makers, regarding participation of older people in activities other than employment has increased (9), thus recognising the full potential of AA to engage older adults in society to be recognised and capitalised upon. However, our study confirms that, in Italy, the structural orientation during the period 2007–2018 (between the Great Recession and the COVID-19 pandemic outbreak) had been a focus on a productivist implementation of AA. Policy goals of extending working lives and postponing retirement age (58) have contributed to a dramatic increase of the AAI *employment* domain score, dragging up the whole AAI score and giving the impression that AA was finally receiving due attention by national and regional policy makers through impactful measures.

Taken together, these findings carry implications for the conceptual debate on positive ageing paradigms. First, they document a measurable gap between AA as a multidimensional concept and AA as an implemented policy outcome, which in the Italian regional experience has approximated narrow productive ageing. Conceptual clarity therefore requires distinguishing between each AA’s conceptual content and its operational realisation. Second, the negative association between growth and social participation cautions against the assumption, implicit in much policy discourse, that economic development automatically advances positive ageing trends across all domains. From a public health perspective, economic growth alone does not lead to the participatory and health-related components of AA, which require deliberate and targeted intervention. Third, because the territorial patterning of these dynamics mirrors long-standing regional inequalities, the opportunity to age actively is itself unequally distributed across Italy, making AA policy a matter of health equity rather than of activation alone.

This study has limitations to consider. The analysis is ecological: regional-level associations cannot be projected onto individual behaviour, and the competing mechanisms of substitution and complementarity are adjudicated only at the aggregate level (56). The number of regional units is small (19), which constrains the models to parsimonious specifications without additional covariates, and the estimates are associations from observational data rather than causal effects. Reverse influences and omitted regional factors—e.g., demographic change, migration, sectoral composition—cannot be excluded, and our descriptive evidence on population dynamics indeed suggests that per capita measures partly reflect demographic rebalancing. The AAI itself has known limitations, including expert-based weighting, a productivist orientation in indicator selection and insensitivity to within-region distribution (8, 27), and some Italian indicators required adaptation from national data sources. Finally, the analysed series ends in 2018: while this provides a clean pre-pandemic baseline, it does not take into account successive evolutions during and after the pandemic years.

Despite its limitations, this study contributes to the literature in the field by highlighting that despite AA is a multidimensional concept, its implementation at the policy level still primarily concerns efforts to promote participation in the labour market, reinforcing the productive ageing approach in its narrowest sense. However, we demonstrate that there are differences when studying the regional level, and that economic development alone cannot be relied upon to deliver positive ageing in its fuller, multidimensional sense. In particular, the results of this study suggest that policy making at the regional governmental level should strive to stimulate older people to increase participation in domains such as volunteering and informal care. Nevertheless, we do not wish to imply that the latter participation should be a forced choice for older individuals, rather, policy makers should ensure that older people, no matter their social and economic conditions, are guided by their individual needs, aspirations and motivations. Additionally, there may be value in exploring AA patterns in other European countries, to consider regional differences that facilitate better understanding of such mechanisms, and to develop common solutions to implementing AA in its multidimensionality. The evidence reported here refers to a single country with a specific regional administrative structure, a familialist welfare arrangement and a pronounced North–South divide; the direction and the size of the associations we document should therefore not be extrapolated to settings with different territorial and welfare arrangements without further evidence.

With regard to Italy, regional government bodies primarily manage programmes and policies affecting the *participation in society* domain (37, 68). Because this AA domain contributed to a much smaller extent than others, there is considerable opportunity for Italian regions, particularly in more economically-disadvantaged areas, to achieve better outcomes by more thoroughly exploring this avenue (69). In this regard, in 2019, the Italian government launched the first national project focused on AA to create a multilevel and co-managed coordination framework between national and regional policy makers, experts, researchers, and stakeholders in civil society). The goal of this national project was to support the design, monitoring, and implementation of AA policies under a multidimensional framework (28). The project recently converged, together with long-term care policy, to the new ageing policy reform (70), which includes among the other things the realisation and implementation of a *National Plan for Active Ageing*, fed by inputs from yearly monitoring AA in all Italian regions, provinces, municipalities, and educational institutions. This may constitute a unique opportunity to carry on a bottom-up approach that can link local needs and preferences with macro-level opportunities in a truly multidimensional framework of AA.

## Data Availability

The raw data supporting the conclusions of this article will be made available by the authors, without undue reservation.
