# Supplementary material for: A productivist drift of active ageing policies in Italy: regional inequality and economic growth in 2007–2018
Source: Front Public Health. 2026 Oct 29;14:1940760. doi: 10.3389/fpubh.2026.1940760 (PMC13758377; doi:10.3389/fpubh.2026.1940760)
Supplement: Supplementary file 1 [file Supplementary_file_1.pdf]

Supplementary Materials

Table A1: Unweighted AAI domain scores (2007-2018)

	Unweighted AAI domain scores							
	2007				2018			
**Region**	**1**	**2**	**3**	**4**	**1**	**2**	**3**	**4**
Piedmont and Aosta Valley	17	8.4	71.6	55.2	31.3	9.4	74.5	55.6
Lombardy	18.4	9.5	71.2	55.3	31.9	11	74.3	58.2
Trentino Alto Adige	21.4	11.8	77.2	58.1	34.7	13.2	76.9	63.6
Veneto	18	10.4	71.3	53.3	31.7	10.5	74.3	57.6
Friuli Venezia Giulia	17.4	8.7	73.5	54.4	32.3	10.5	75.9	59
Liguria	20.4	6.6	72.6	55.3	33.4	7.6	74.6	56.6
Emilia Romagna	22.4	9.6	71.7	53.7	35.2	10.9	74.5	56.7
Tuscany	20.8	8.4	71.5	55.2	35.2	9.3	74.4	58.4
Umbria	19.9	7.4	71	53.6	32.2	9.7	69.4	55.6
Marche	21.1	6.6	70.6	52.9	33.7	9.4	73	54.9
Latium	21.3	6.3	68.5	52.9	33.4	8.7	71.8	58.1
Abruzzo	19.4	6.5	68.4	52.5	31.6	8.2	72	55.6
Molise	17.9	6.1	69.4	52.4	30.7	6.8	71.5	58.7
Campania	18.6	6.3	65.3	49.2	26.1	6	67.9	51.5
Apulia	16.8	6.5	65.7	49.5	24.4	8.7	68.5	53.1
Basilicata	20.6	6.9	67.2	50.4	30.4	9.4	69.9	54.9
Calabria	19.1	5.3	68.6	49.2	25.2	7.9	68.7	51.9
Sicily	17.4	5.8	66.9	47.5	23.8	7.1	67.9	51.2
Sardinia	17.3	8.7	69.3	51.1	29.1	8.8	72.2	55.1
Italy	19	7.8	70	52.9	30.6	9.2	72.3	55.9

Legend: 1 = Employment; 2 = *Participation in society*; 3 = Independent, healthy and secure living; 4 = Capacity and enabling environment for active ageing

Table A2: Differences between the UNECE AAI and the Italian AAI

**AAI indicator**	**AAI-EU**	**AAI IT**
**Employment**		
Employment rate 55-59	Did you do any paid work in the 7 days ending Sunday the [date], either as an employee or as self-employed? (**Yes**)<br><br>Even though you were not doing paid work, did you have a job or business that you were away from in the week ending Sunday the [date] (and that you expect to return to)? (**Yes**)	Same
Employment rate 60-64	See AAI EU wording 1.1	Same
Employment rate 65-69	See AAI EU wording 1.1	Same
Employment rate 70-74	See AAI EU wording 1.1	Same
Participation in society		
Voluntary activities	How often (55+) did you do unpaid voluntary work through the following organizations in the last 12 months? [list of organizations] (**every week**)	Same
Care to children and grandchildren	In general, how often are you involved in any of the following activities outside of paid work? a. Caring for and/or educating your children b. Caring for and/or educating your	Did you (55+) provide some of the following types of unpaid help to individuals (relatives and not) that do not live with you,

	grandchildren. (**Every day; Several days a week; Once or twice a week**)	in the last four weeks? (multi-response, concerning 11 possible types of help): care and assistance to children.
Care to older adults	In general, how often are you involved in any of the following activities outside of paid work? d. Caring for disabled or infirm family members, neighbours or friends under 75 years old e. Caring for disabled or infirm family members, neighbours or friends aged 75 or over. (**Every day; Several days a week; Once or twice a week**)	Did you (55+) provide some of the following types of unpaid help to individuals (relatives and not) that do not live with you, in the last four weeks? (multi-response, concerning 11 possible types of help): care and assistance to adult individual (help in washing, dressing, eating, etc.).
Political participation	Over the last 12 months, have you (55+)…? A) Attended a meeting of a trade union, a political party or political action group; or B) Attended a protest or demonstration; or C) Signed a petition, including an e-mail or online petition; or D) Contacted a politician or public official (other than routine contact arising from use of public services) (**Yes**)	Did you A) participate in meetings of a trade union, political party, ecological associations, for civil rights or for peace; B) or in a rally or a protest demonstration in the last 12 months? (**Yes**)
**Independent, healthy and secure living**		
Physical exercise	How frequently do you (55+) do each of the following? c. Take part in sports or physical exercise. (**every day or almost every day**)	Do you (55+) practice one or more type of sports continuously, or do you do physical activity (e.g. walking at least two kilometres, swimming, cycling, or other) one or more times a week? (**Yes**)
Access to health services	The indicator refers to respondents (55+) who say that there was no occasion when the person really needed medical or	Same

	dental examination or treatment but was not able to receive it.	
Independent living	Percentage of people aged 75 and over who live in a single-person household or who live as a couple (2 adults with no dependent children).	Same
Financial security – Relative median income	The relative median income ratio is defined as the ratio of the median equivalised disposable income of people aged 65 and over to the median equivalised disposable income of those aged below 65. Household disposable income is established by summing up all monetary incomes received from any source by each member of the household (including income from work, investment and social benefits) – plus income received at the household level – and deducting taxes and social contributions paid. In order to reflect differences in household size and composition, this total is divided by the number of 'equivalent adults' using a standard (equivalence) scale, the so-called 'modified OECD' scale, which attributes a weight of 1 to the first adult in the household, a weight of 0.5 to each subsequent member of the household aged 14 and over, and a weight of 0.3 to household members aged less than 14. The resulting figure is called equivalised disposable income and is attributed to each member of the household.	Same
Financial security – No poverty risk	Percentage of people aged 65 and over who are not at risk of poverty (people at risk of poverty are defined as those with an equivalised disposable income after social transfers below the at-risk-of-poverty threshold, which is set at 50% of the national	Same

	median equivalised disposable income after social transfers).	
Financial security – No material deprivation	Data on the material items mentioned in the following is collected using a direct question at the household level. Severe material deprivation refers to a state of economic and durable strain of individuals 65+, defined as the enforced inability (rather than the choice not to do so) to afford at least four out of the following nine items: 1) to pay their (a) rent, (b) mortgage or (c) utility bills; 2) to keep their home adequately warm; 3) to face unexpected expenses; 4) to eat meat or proteins regularly; 5) to go on holiday; 6) a television set; 7) a washing machine; 8) a car; 9) a telephone.	Same
Physical safety	'How safe do you (55+) – or would you - feel walking alone in this area (Respondent's local area or neighbourhood) after dark? Do – or would – you feel' (**very safe; safe**)	Does the living area of your family have crime risks? (**few; not at all**).
Lifelong learning	Did you attend any courses, seminars, conferences, or receive private lessons or instructions within or outside the regular education system within the last 4 weeks? (**yes**)	Same
**Capacity and enabling environment for active ageing**		
Remaining life expectancy at age 55	Remaining life expectancy (RLE) at 55 divided by 50 to calculate the proportion of life expectancy achievement in the target of 105 years of life expectancy.	Same
Share of healthy life expectancy at	The indicator is calculated applying the Sullivan method based on the life tables and an indicator of self-perceived long-standing limitations in usual activities due to health problems.	Same

age 55	The indicator is: Limitation in activities because of health problems [General activity limitation: Limitation in activities people usually do because of health problems for at least the past six months]. (**yes, strongly limited; yes, limited**).	
Mental well-being	Over the last two weeks (55+): 1 - I have felt cheerful and in good spirits 2 - I have felt calm and relaxed 3 - I have felt active and vigorous 4 - I woke up feeling fresh and rested 5 - My daily life has been filled with things that interest me Response categories of each of these five survey questions are: 1. All of the time 2. Most of the time 3. More than half of the time 4. Less than half of the time 5. Some of the time 6. At no time The raw score is calculated by reversing the value order of the variable, and then totaling the figures of the five answers. The raw score is converted so as to range from 0 to 25, 0 representing worst possible and 25 representing best possible quality of life. According to WHO, a raw score below 13 indicates poor wellbeing and is an indication for testing for depression under the Major Depression (ICD10) Inventory.	In the last 4 weeks you (55+) felt: 1) happy 2) calm and/or peaceful 3) full of energy 4) very down to earth (scale reversed) 5) discouraged and sad (scale reversed) Response categories of each of these five survey questions are: 1. Always = 5 2. Almost always = 4 3. A lot of time = 3 4. Some of the time = 2 5. Almost never = 1 6. Never = 0 The range is from 0 to 25, 0 representing worst possible and 25 representing best possible quality of life, and it has been converted to range from 0 to 100.
Use of ICT	How often on average have you (55-74) used the Internet in the last 3 months? (**every day or almost every day; at least once a week**)	How often on average have you (55-74) used the Internet in the last 12 months (3 months in 2007)? (**every day; almost every day; once a week**)
Social connectedness	How often do you (55+) socially meet with friends, relatives or colleagues? (**once a week; several times a week; every day**).	Same

Educational attainment	% of 55-74 with upper secondary or tertiary educational attainment (ISCED 3 or higher)	Same

Table A3: AAI domains change and regional economic growth by NUTS 2 regions (Pooled OLS: 2007-2009/2009-2012/2012-2018). Sensitivity checks.

	(1)<br>Δ PARTICIPATION<br>(CAL out)	(2)<br>Δ PARTICIPATION<br>(CAL & MOL out)	(3)<br>Δ INDEP. LIVING<br>(3.7 out)	(4)<br>Δ CAPACITY<br>(4.4 out)
Panel A				
$\Delta PCGDP_{r,t_0-t_1}$	-0.456*	-0.449*	-0.360	0.397
	(0.196)	(0.198)	(0.336)	(0.363)
Period FE	yes	yes	yes	yes
Region FE	yes	yes	yes	yes
N	54	51	57	57
R^2	0.582	0.589	0.132	0.286

Notes. Pooled OLS regressions. Robust standard errors clustered by region. Models weighted by regions' share of national population in 2007. Columns 1 and 2 leave the Calabria region and the Calabria and Molise regions out, respectively. Columns 3 and 4 leave indicators 3.7 and 4.4 out, respectively. For data availability reasons, Piedmont and Aosta Valley regions are grouped in a single region. * p<0.05, ** p<0.01, *** p<0.001. Wild cluster bootstrap-t p-values for the coefficient on Δ GDP per capita, computed with the null imposed and Webb weights (9,999 replications), are 0.043, 0.049, 0.264 and 0.300 in columns 1 to 4, and the corresponding 95% confidence sets are [−0.768; −0.020], [−0.760; −0.003], [−0.989; 0.330] and [−0.268; 1.161].
